# Physiotherapy Rehabilitation Post Total Hip Replacement in the Case of Avascular Necrosis of the Femur: A Case Report

**DOI:** 10.7759/cureus.33465

**Published:** 2023-01-06

**Authors:** Chitrakshi A Choubisa, Akanksha R Hege, Pratik Phansopkar

**Affiliations:** 1 Department of Musculoskeletal Physiotherapy, Ravi Nair Physiotherapy College, Datta Meghe Institute of Medical Sciences, Wardha, IND

**Keywords:** functional independence, rehabilitation, physical therapy, total hip replacement, avascular necrosis

## Abstract

Avascular necrosis of the femur is an irreversible and painful disorder in which the epiphyseal bone suffers from ischemia necrosis owing to an interruption in blood flow to the femoral head, resulting in bone destruction. Later, it leads to osteoarthritis of the hip joint. Here, we present the case of a 35-year-old male who came with a complaint of pain on the left side of the hip region for the past 15 days. Since the patient tested positive for COVID-19, he was quarantined. An X-ray was carried out once the quarantine period was completed, which revealed avascular necrosis of the left femoral head. He was advised to have a total hip replacement and underwent the surgery. After one month, the patient started experiencing pain on the right side of the hip region. He visited the rural hospital, where an X-ray was carried out, which revealed avascular necrosis of the right femoral head. For reducing pain and improving functional independence and quality of life postoperatively, a well-planned physiotherapy protocol was incorporated, which included lower limb and pelvic floor strengthening exercises and a balance training program. The Numerical Pain Rating Scale and Harris Hip Score have been used as outcome measures to demonstrate the efficacy of the treatment.

## Introduction

Avascular necrosis is an irreversible and painful disorder in which the epiphyseal bone suffers from ischemia necrosis owing to an interruption in blood flow to the femoral head, resulting in bone destruction [1]. It is also known as osteonecrosis [2]. In more than 75% of cases, avascular necrosis affects the femoral head, which often affects people under the age of 50 and frequently results in femoral head collapse and later hip arthritis [1]. Although the epidemiology of avascular femoral head necrosis is yet unknown, several risk factors must be investigated, including COVID-19 patients receiving corticosteroid therapy [3], hypercholesterolemia, sickle cell disease, alcohol misuse, and organ transplant management [1].

Medical treatment for this condition is based on the Ficat and Arlet classification of the disease stage. In stages I and II, the joint surface is typically intact; therefore, conservative treatment with pharmacological therapy is indicated, but stages III and IV, which are more advanced, necessitate core decompression [4], osteotomy, and complete hip arthroplasty [1,5]. However, more recent therapeutic approaches have been created to administer stem cells to the necrotic areas to prevent fracture and collapse by repairing the femoral head's structural integrity [6]. Physiotherapy, along with medical and surgical management, can prove effective in this condition. Physical therapy can help to alleviate symptoms and improve functional independence and quality of life in people with this condition [7]. After surgical care for avascular necrosis, physiotherapy interventions are essential in the patient's rehabilitation. Physiotherapy is useful in minimizing postoperative discomfort in this ailment [8].

## Case presentation

Patient’s information

**Figure 1:**
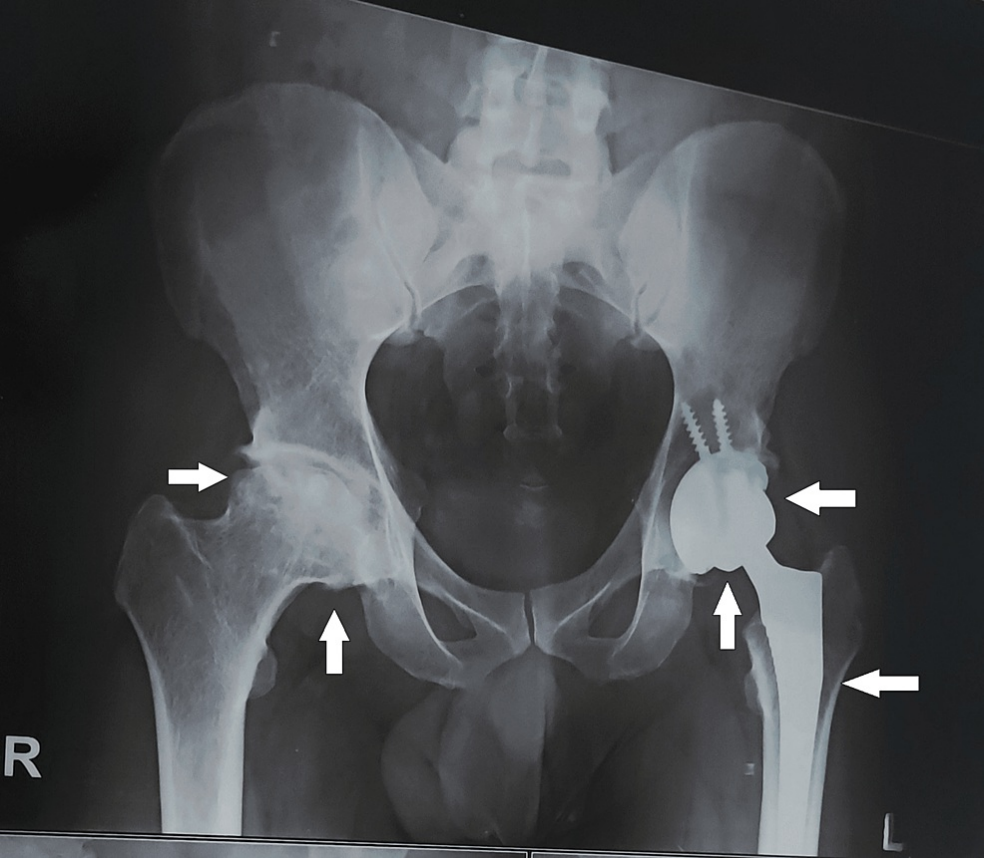
Anteroposterior view of an X-ray of the pelvis showing the destruction of the femoral head on the right side and partial hip replacement on the left side.

**Figure 2:**
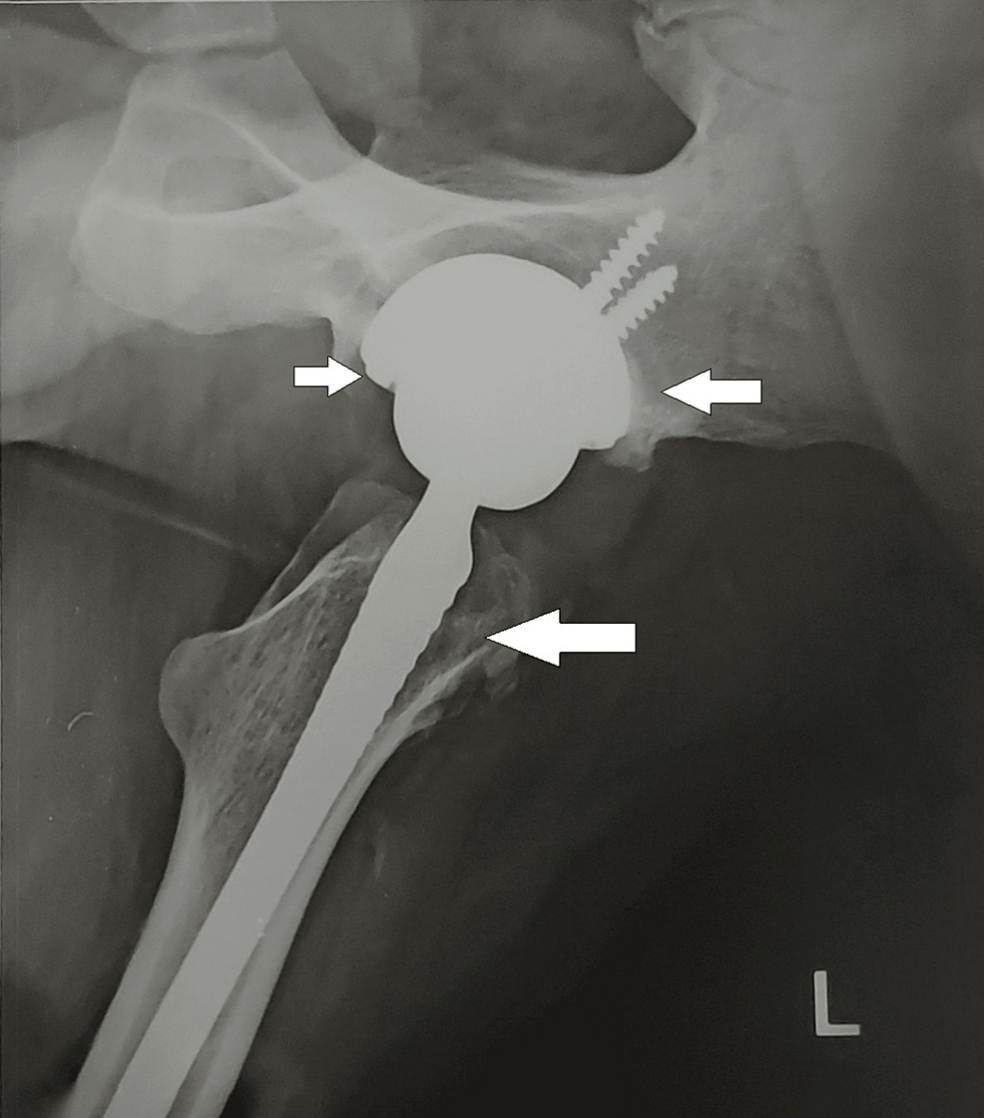
Lateral view of an X-ray of the pelvis showing total hip replacement on the left side.

**Figure 3:**
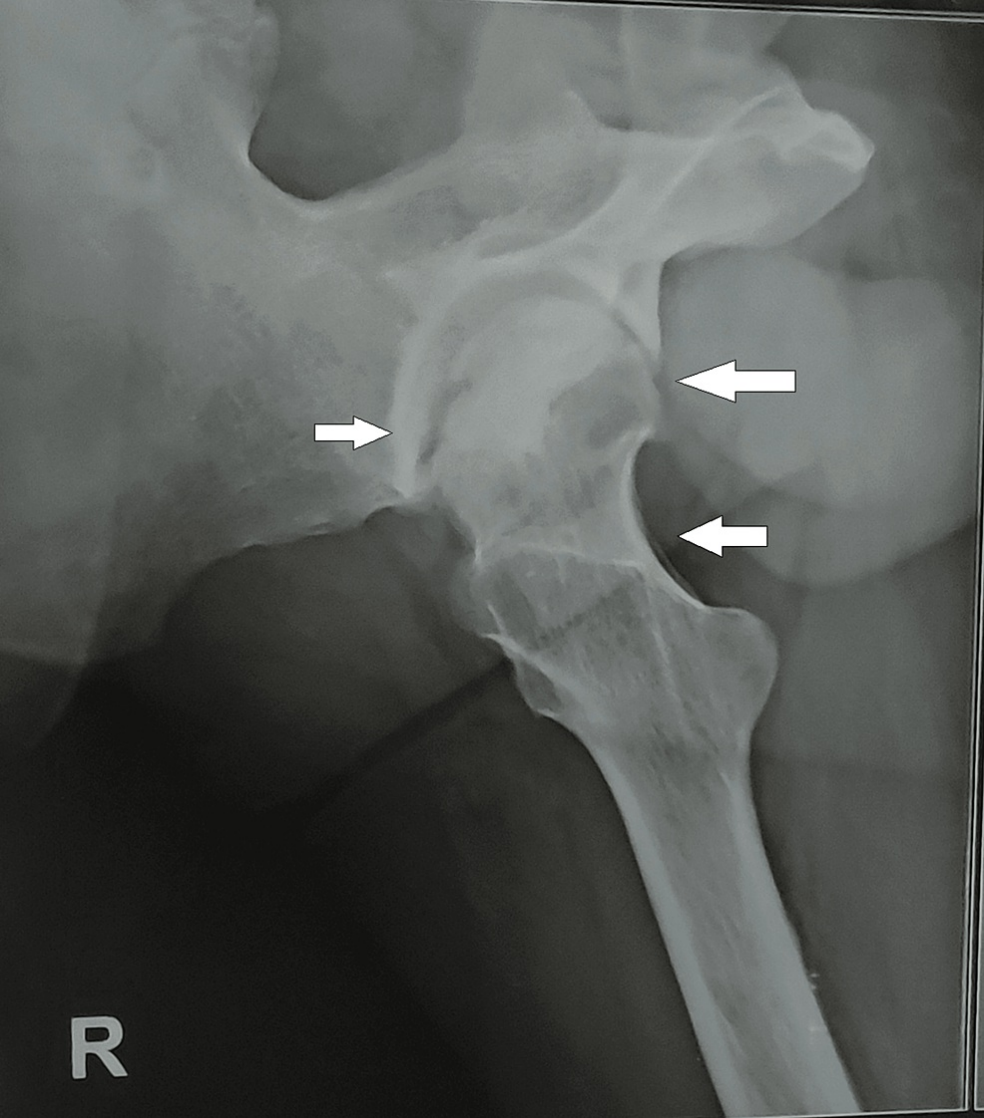
Lateral view of an X-ray showing the destruction of the femoral head on the right side.

A 35-year-old male, an automobile mechanic by occupation, came with the complaint of pain on the left side of the hip region for the past 15 days along with difficulty walking. The patient was apparently alright one month ago when he tested positive for COVID-19. While he was quarantined, he started experiencing pain on the left side of the hip region, which was gradual in onset and progressive in nature, aggravated by walking, squatting, and prolonged standing, and relieved by rest. After his recovery from COVID-19, he visited the rural hospital, where an X-ray was done, which revealed avascular necrosis of the left femoral head. The patient was advised to have a total hip replacement and underwent the surgery. After one month, the patient started experiencing pain on the right side of the hip region. He visited the rural hospital, where an X-ray was carried out, which revealed destruction of the femoral head. He was diagnosed with avascular necrosis of the right femoral head (Figures 1-3). Physiotherapy rehabilitation was further started.

Clinical findings

Prior to the assessment, informed consent was taken, and the patient was positioned in the supine lying position. On general examination, the patient was conscious, cooperative, well oriented to time, place, and person, hemodynamically stable, and afebrile. He was ectomorphic, with a BMI of 17.8 kg/m^2^. On observation, the patient was having the antalgic type of gait with both hips externally rotated. The limb length on the left side (operated side) was shorter than on the right side. On inspection, the surgical scar was present on the left side. On palpation, the local temperature was not increased, grade-2 tenderness was present on the left side, and grade-3 tenderness was present on the right side. The hip range of motion (ROM) was observably reduced, and all hip movements were painful. The hamstrings and abductor muscles were tight on both sides.

Therapeutic intervention

Physiotherapy rehabilitation protocol was inculcated (Table 1).

**Table 1:** Illustration of physiotherapy protocol.

Goals	Physiotherapy intervention	Intervention regime
To reduce pain and swelling	Cryotherapy	Cryotherapy: 10 minutes, two times a day
Positioning	Pillow should be placed between both legs to avoid adduction of the hip	Should be done while lying and long sitting on the bed
To improve hip mobility and strengthening of hip muscles	Straight leg raise (Figure 4)	10 repetitions x 1 set, progressed by adding ½ kg weight on the second week and 1 kg weight on the third and fourth weeks
	Hip abduction in side lying (Figure 5)	
	Bridging	10 repetitions, with five-second hold initially for the first week, progressed to 10-second hold for the second, 15 repetitions x 1 set for the third and fourth weeks
To improve the strength of bilateral lower extremities and core	Pelvic tilts on a Swiss ball	10 repetitions x 1 set initiated from the second week, progressed to 15 repetitions x 1 set for the third and fourth weeks
	Dynamic quadriceps	
	Squatting	
	Lunges	
To improve balance and proprioception	Single leg stance (Figure 6)	10 repetitions x 1 set initiated from the third week, 15 repetitions x 1 set for the fourth week
	Standing and squatting on a Bosu ball (Figures 7 and 8)	5 repetitions x 1 set on the third week, progressed to 10 repetitions x 1 set on the fourth week
To improve gait and stair climbing	Hall ambulation	Two rounds of 15 m hallway initiated on the third week, progressed to four rounds of 15 m hallway on the fourth week
	Step climbing and steeping down on a Bosu ball (Figure 9A and B)	10 repetitions x 1 set initiated on the third week, progressed to 10 repetitions x 2 sets on the fourth week

**Figure 4:**
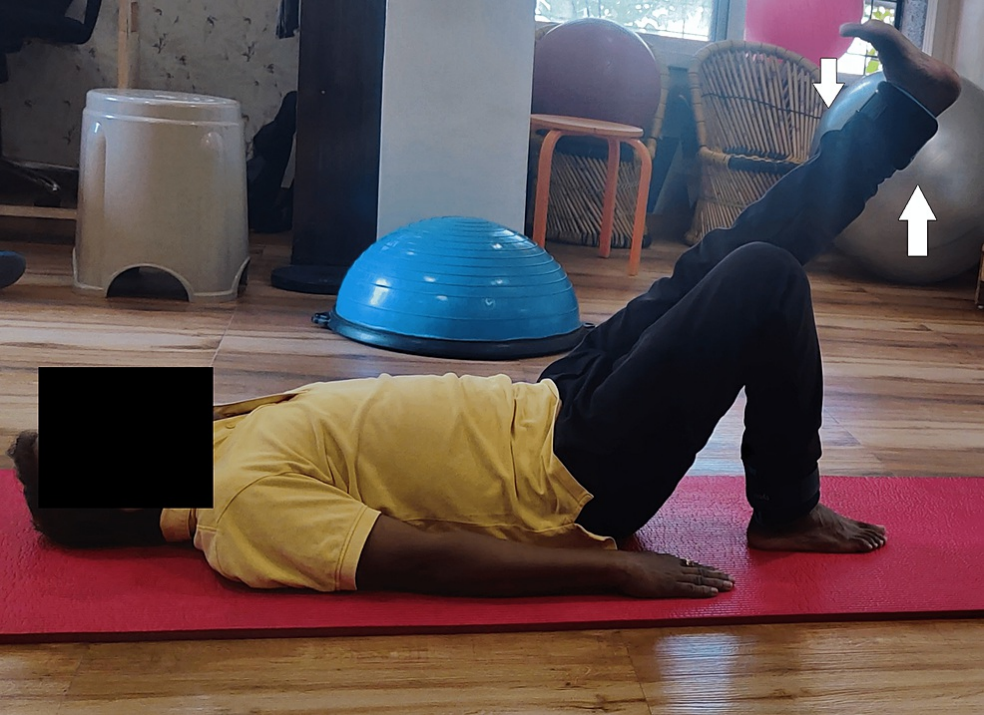
Patient performing straight leg raises in the supine lying position.

**Figure 5:**
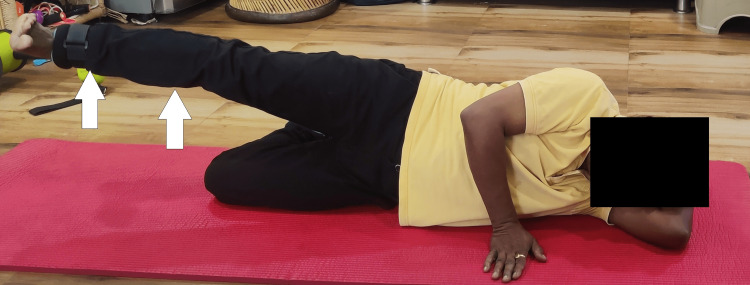
Patient performing hip abduction in the side lying position.

 The intervention included cryotherapy, straight leg raises, and hip abduction (Figures 4 and 5).

**Figure 6:**
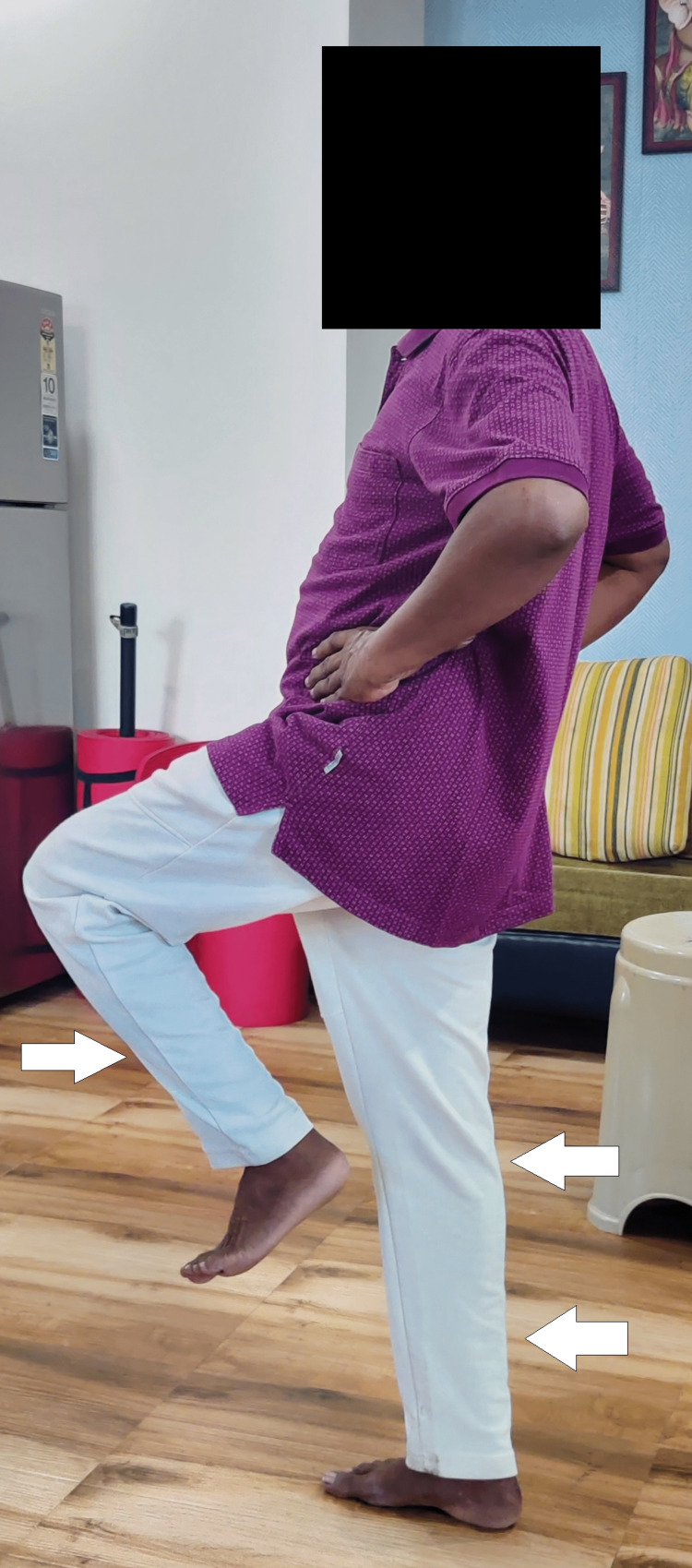
Single leg stance performed by the patient.

Figure 6 shows a patient performing a single leg stance.

**Figure 7:**
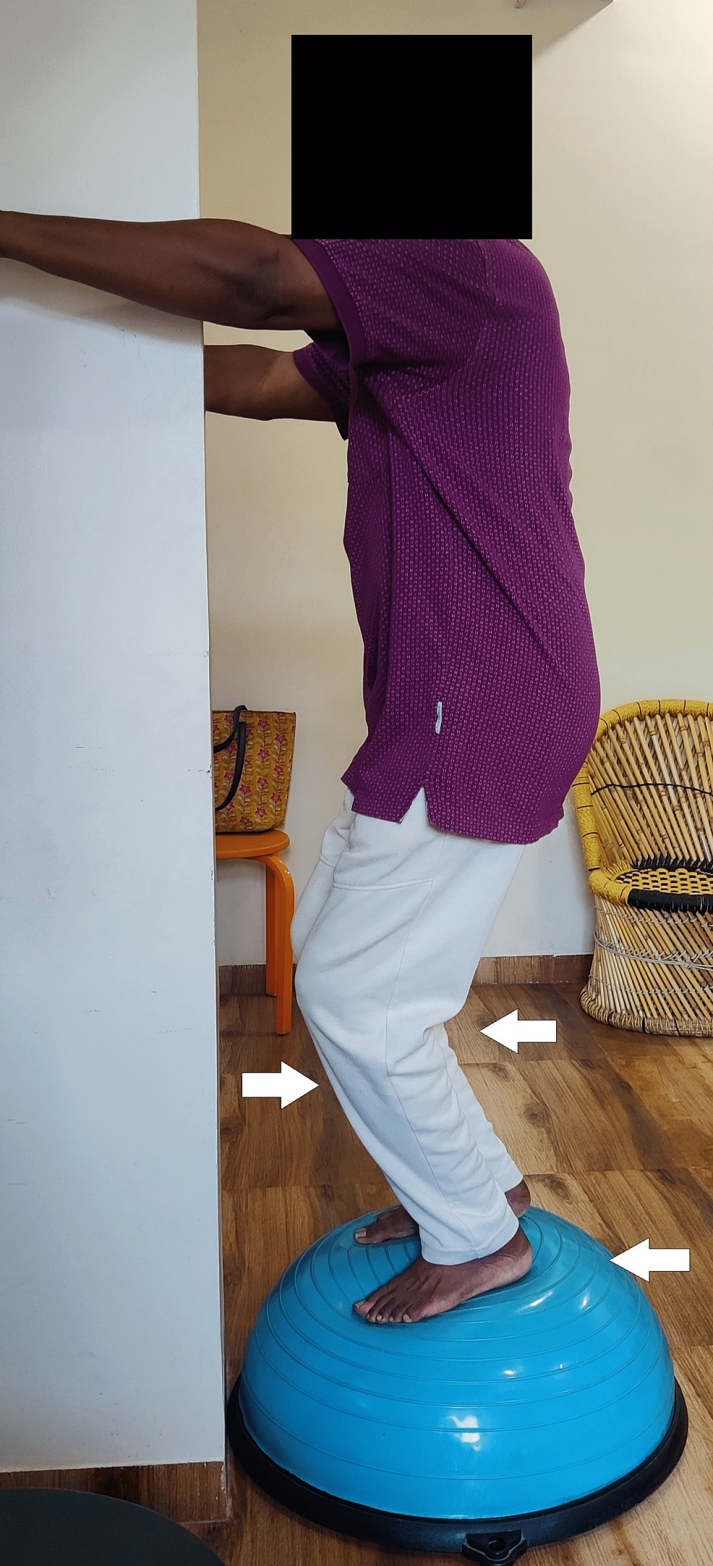
Patient performing squatting on a Bosu ball.

**Figure 8:**
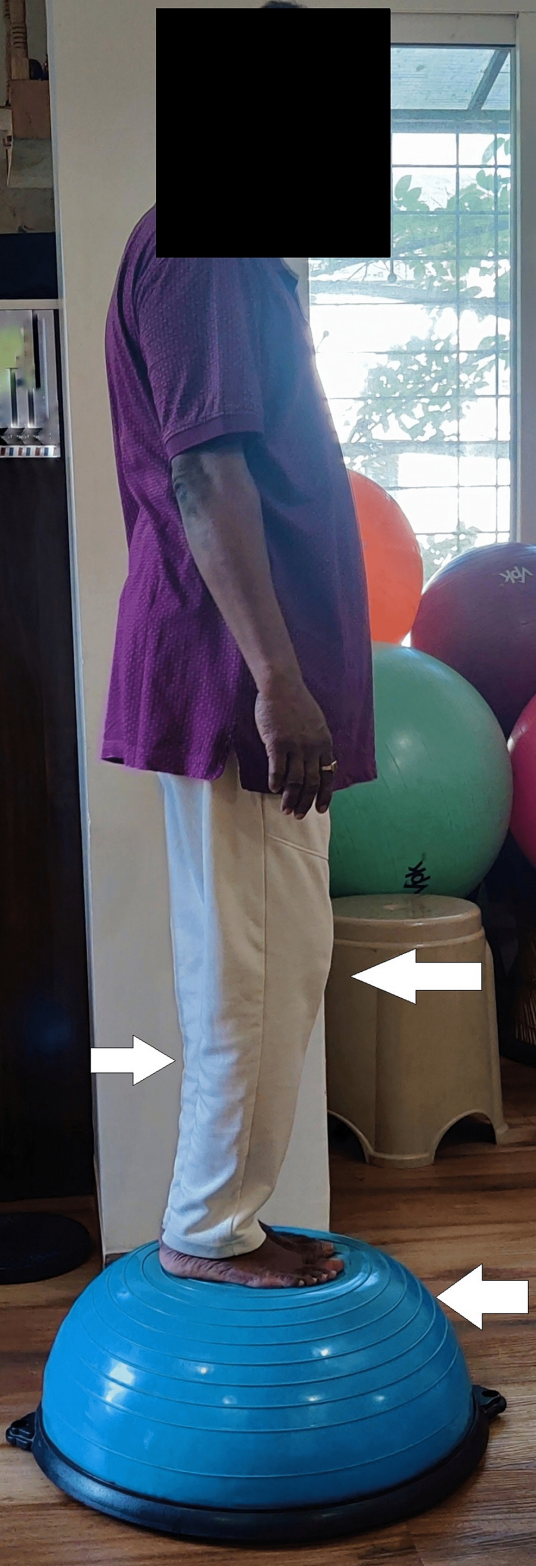
Patient standing on a Bosu ball.

Figures 7 and 8 show a patient squatting and standing on a Bosu ball, respectively.

**Figure 9:**
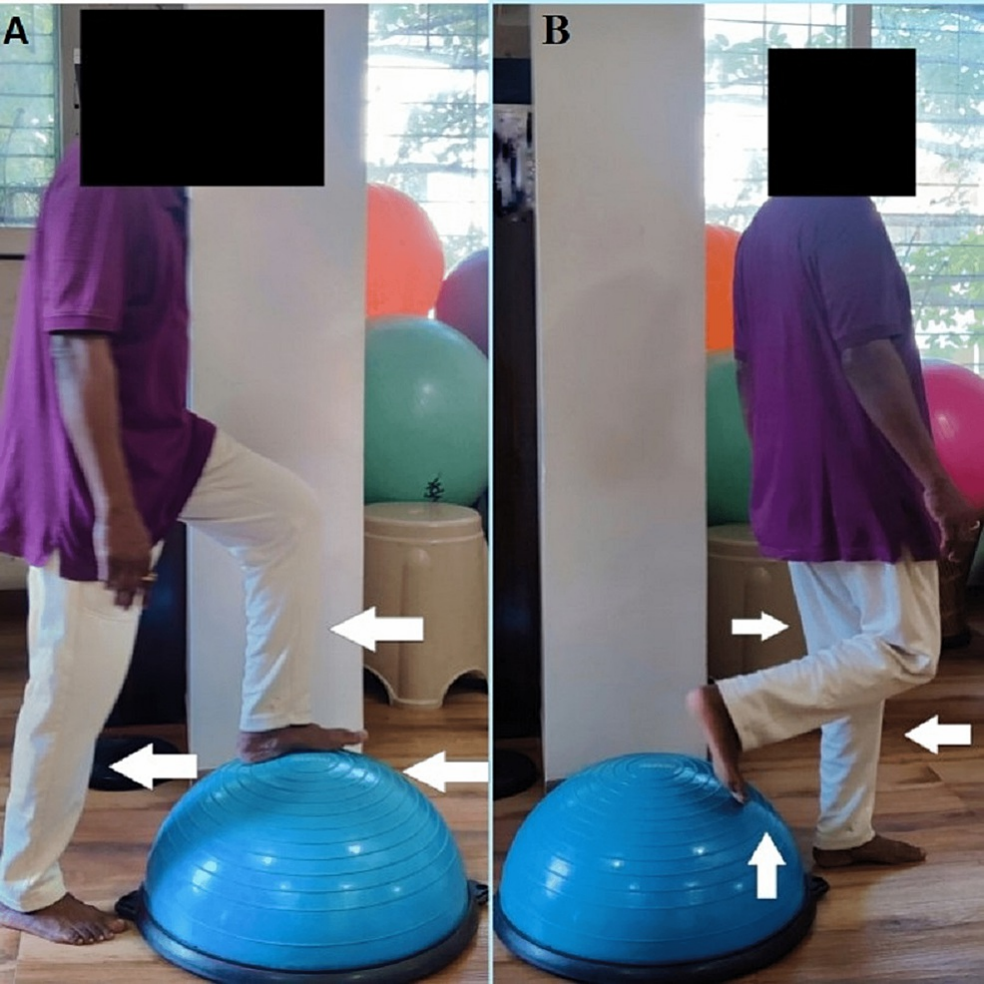
(A) Patient performing step climbing on a Bosu ball. (B) Patient stepping down from a Bosu ball.

Figure 9A and B shows a patient stepping up and stepping down on a Bosu ball. 

Follow-up and outcome

A well-planned physiotherapy interventional protocol was initiated. Follow-up was taken once a week for four weeks. Table 2 illustrates outcome measure scores.

**Table 2:** Outcome measure scores.

Scale	First week	Second week	Third week	Fourth week
Numerical Pain Rating Scale	09/10	09/10	08/10	07/10
Harris Hip Score	50/100	60/100	60/100	70/100

## Discussion

Avascular necrosis is a degenerative bone disease that causes the loss of bone cellular components because of a breakdown in the subchondral blood supply. Osteonecrosis, aseptic necrosis, and ischemic bone necrosis are other names for this condition. Usually, it affects long bones' epiphyses at weight-bearing joints [2,7]. 

In this case, the patient has pain over the left hip region along with difficulty walking, stair climbing, and carrying out activities of daily living. He was diagnosed with avascular necrosis of the femur and managed with a total hip replacement. A concomitant and well-planned physiotherapy intervention protocol was formulated, which included simpler exercises initially, followed by strengthening exercises. Protocol started with hip ROM exercises and progressed to balance training and resistance exercises. According to a study by Saklecha et al., patients with avascular necrosis benefited from the work of a multidisciplinary team that used medicinal, surgical, and rehabilitative physiotherapy techniques to reduce pain, improve muscular performance, and make patients functionally independent [7].

A study found that the total hip replacement rehabilitation program enhanced hip motion and muscle strength, along with the patient's walking ability, Harris Hip Score, Pain Disability Index, and Numerical Pain Rating Scale [5]. According to Kariya et al., physiotherapy interventions, including quadriceps strengthening and bedside activity training, were effective in postoperative avascular necrosis for early restoration of strength, ROM, and functional activities [8]. Patients with ischemic necrosis can endure a well-designed physical therapy regimen, and it can help them recover their physical function more quickly following total hip replacement [9]. Karim's study on necrosis of the femoral head found that an exercise regimen helped patients recover promptly [10]. According to Domínguez-Navarro et al., following total joint replacement, balance and proprioceptive impairments are usually permanent, limiting functionality and causing patients to move differently and have trouble walking and maintaining posture control; therefore, balance and proprioceptive training is considered a crucial aspect of rehabilitation [11]. This case report's objectives are to emphasize the value of physical therapy rehabilitation and to provide a comprehensive plan for the care of total hip replacement in patients with avascular necrosis.

## Conclusions

The post-hip replacement therapy is successful, resulting in appreciable improvements in physical function and general well-being. This case report suggests that well-organized physiotherapy management along with medical and surgical management has proven to be immensely effective in reducing pain and improving functional independence and quality of life of the patient with avascular necrosis of the femoral head followed by a total hip replacement. However, complete recovery had not been achieved, but the majority of the restorative aims had been fulfilled.
